# Supplementary figures and images for: Changes in airway diameter and mucus plugs in patients with asthma exacerbation
Source: PLoS One. 2020 Feb 27;15(2):e0229238. doi: 10.1371/journal.pone.0229238 (PMC7046273; doi:10.1371/journal.pone.0229238)

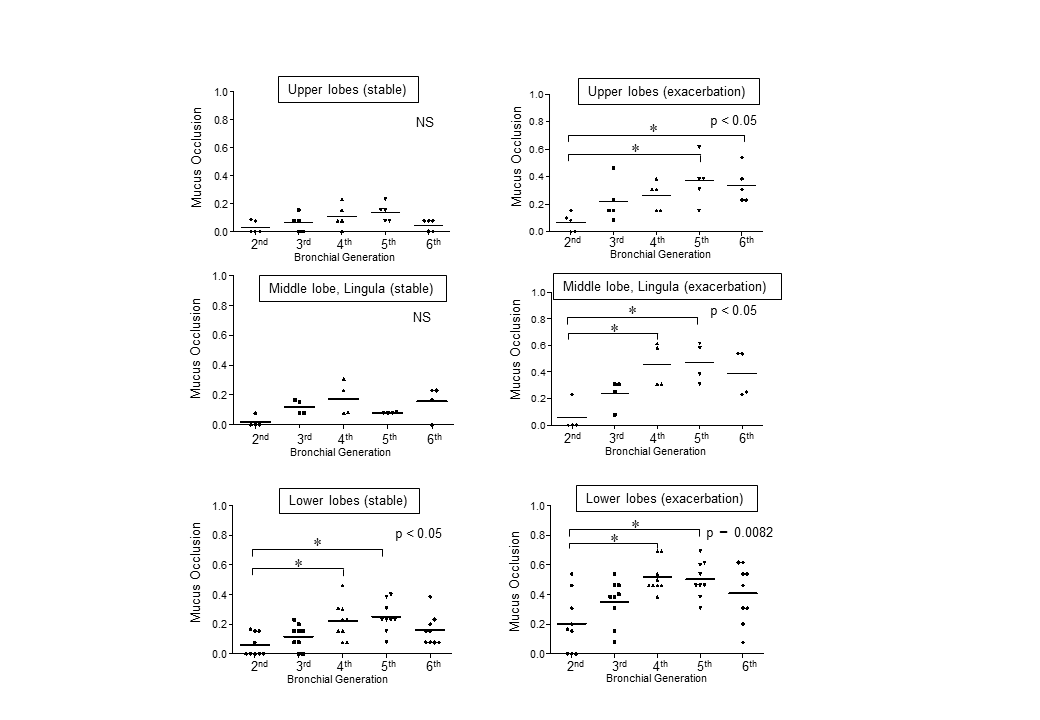

Supplement: S1 Fig — Mucus occlusions were measured by HRCT using curved MPR software. Mucus occlusions were counted per airway generation in all segments of the lung for each subject. Airway segment data were grouped by lobes and by airway generation for analysis. Each point represents the ratio of mucus-occluded compartments in 13 patients for each segment. (TIF) [file pone.0229238.s001.tif]

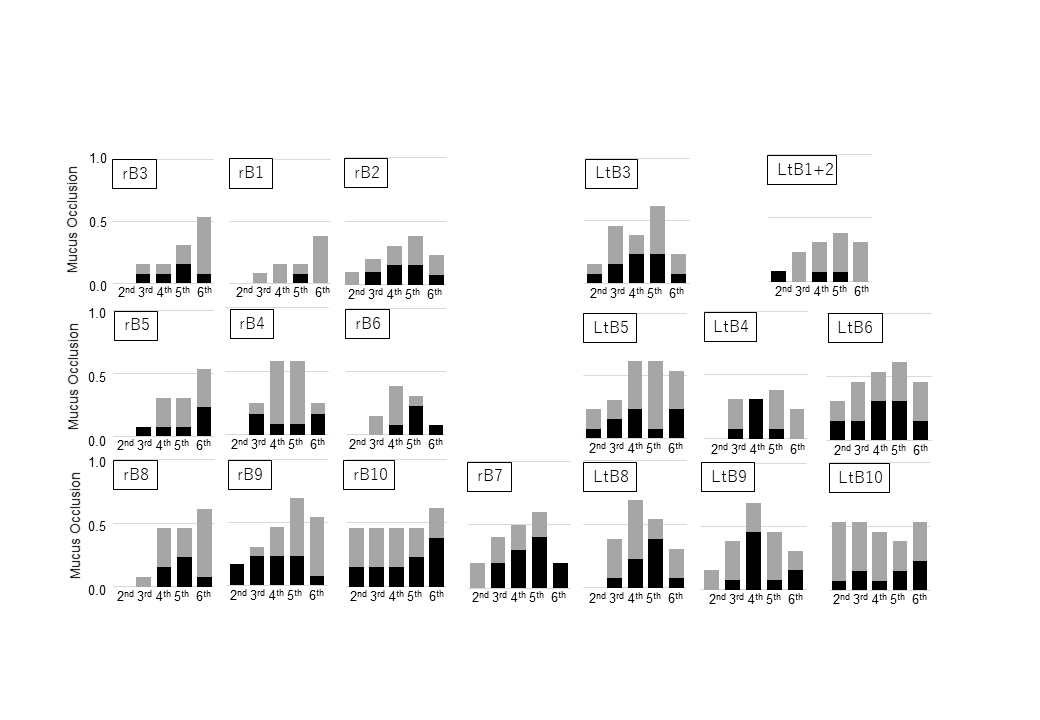

Supplement: S2 Fig — Mucus occlusion (mucus plug) was measured by HRCT for each subject at suspended end-inspiratory volume using curved MPR software. Airway segment data per airway generation are expressed. The black bar represents the ratio of mucus occlusion in the stable phase, and the black bar plus gray bar represents the ratio of mucus occlusion during asthma exacerbation. (TIF) [file pone.0229238.s002.tif]
